# Interaction between NBS1 and the mTOR/Rictor/SIN1 Complex through Specific Domains

**DOI:** 10.1371/journal.pone.0065586

**Published:** 2013-06-06

**Authors:** Jian-Qiu Wang, Jian-Hong Chen, Yen-Chung Chen, Mei-Yu Chen, Chia-Ying Hsieh, Shu-Chun Teng, Kou-Juey Wu

**Affiliations:** 1 Institute of Biochemistry and Molecular Biology, National Yang-Ming University, Taipei, Taiwan; 2 Institute of Aging Research, Department of Basic Medical Science, School of Medicine, Hangzhou Normal University, Hangzhou, China; 3 Institute of Microbiology, College of Medicine, National Taiwan University, Taipei, Taiwan; University of Chicago, United States of America

## Abstract

Nijmegen breakage syndrome (NBS) is a chromosomal-instability syndrome. The NBS gene product, NBS1 (p95 or nibrin), is a part of the Mre11-Rad50-NBS1 complex. SIN1 is a component of the mTOR/Rictor/SIN1 complex mediating the activation of Akt. Here we show that NBS1 interacted with mTOR, Rictor, and SIN1. The specific domains of mTOR, Rictor, or SIN1 interacted with the internal domain (a.a. 221-402) of NBS1. Sucrose density gradient showed that NBS1 was located in the same fractions as the mTOR/Rictor/SIN1 complex. Knockdown of NBS1 decreased the levels of phosphorylated Akt and its downstream targets. Ionizing radiation (IR) increased the NBS1 levels and activated Akt activity. These results demonstrate that NBS1 interacts with the mTOR/Rictor/SIN1 complex through the a.a. 221–402 domain and contributes to the activation of Akt activity.

## Introduction

Nijmegen breakage syndrome (NBS) is a chromosomal-instability syndrome with the manifestation of cancer predisposition, radiosensitivity, microcephaly, and growth retardation [1]–[3]. The NBS gene product, NBS1 (p95 or nibrin), is a part of the Mre11-Rad50-NBS1 complex that is essential for DNA double strand break repair [1], [2]. NBS1 carries out its checkpoint functions through the phosphorylation by ataxia-telangiectasia mutated (ATM) protein after ionizing radiation [4]–[6]. We previously demonstrated that c-MYC, an oncoprotein, directly activates the expression of NBS1 [7]. The function of NBS1 related to proliferation is demonstrated by the phenotypes of diminished expansion of the inner cell mass of mutant blastocysts (Nbs1 null) and cellular proliferation defects in Nbs1**^m/m^** mouse embryonic fibroblasts [8]–[10]. Overexpression of NBS1 induces transformation activity through interacting with the p110 subunits of phosphoinositide 3-kinase (PI 3-kinase) to activate PI 3-kinase activity [11], [12], indicating that NBS1 overexpression is an oncogenic event. In head and neck squamous cell carcinoma patients, increased NBS1 expression is a prognostic factor of aggressive head and neck cancer [13]. All these results indicate that NBS1 overexpression may play an important role in tumorigenesis.

Akt is a well-known downstream target of PI 3-kinase [14], [15]. Akt is activated through phosphorylation at Thr-308 by phosphoinositide-dependent protein kinase 1 followed by phosphorylation at Ser-473 by the mTOR/Rictor (mTORC2) complex to achieve full activation [16], [17]. Activated Akt then regulates a wide range of target proteins that control cell proliferation (e.g. GSK-3β, Foxo1/3a), survival (e.g. BAD), and cell growth (e.g. mTOR) [15]. Akt regulates critical processes of tumorigenesis and plays an important role in oncogenesis [16]. Altered expression or mutation of many components of the PI3K/Akt pathway has been implicated in human cancer [15], [18]. Recently, a new member of the mTORC2 complex, SIN1, was identified [19]–[21]. SIN1 is critical to maintain the mTORC2 complex and regulate Akt kinase activity and substrate specificity [19]–[21]. Other example such as TSC1-TSC2 complex was shown to associate with the mTORC2 complex and activate Akt kianse activity (Akt Ser-473 phosphorylation) [22]. However, it remains to be explored whether other protein could participate in the mTORC2 complex to induce Akt kinase activity.

In this report, we demonstrate that NBS1 interacts with the mTOR/Rictor/SIN1 complex using a specific domain (a.a. 221–402). The domain in mTOR, Rictor, or SIN1 that interacts with NBS1 is also mapped. Knockdown of NBS1 decreases Akt kinase activity. IR increased the levels of NBS1 and phosphorylated Akt. These results indicate that NBS1 is a critical component of the mTOR/Rictor/SIN1 complex to activate Akt kinase.

## Results

### Interaction between NBS1 and the Components of the mTOR/Rictor/SIN1 Complex

**Figure 1:**
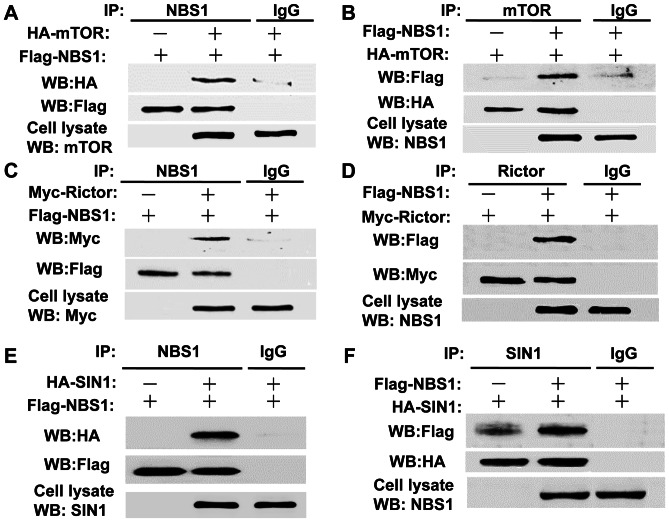
Interaction between NBS1 and the mTOR/Rictor/SIN1 complex. **A & B.** Co-immunoprecipitation assays showed the interaction between NBS1 and mTOR in 293T cells overexpressing both proteins. **C & D.** Co-immunoprecipitation assays showed the interaction between NBS1 and Rictor in 293T cells overexpressing both proteins. **E & F.** Co-immunoprecipitation assays showed the interaction between NBS1 and SIN1β in 293T cells overexpressing both proteins.

We previously demonstrated that NBS1 interacted with the p110α subunit of the PI 3-kinase to induce PI 3-kinase/Akt activity [12]. However, it is possible that NBS1 may also interact with Akt to induce Akt activity. To test whether there is interaction between NBS1 and the mTOR/Rictor/SIN1 complex that is responsible for the activation of Akt activity, co-immunoprecipitation assays were performed between NBS1 and each component of the mTOR/Rictor/SIN1 complex. The results showed that the anti-NBS1 antibody pulled down mTOR in 293T cells overexpressing both NBS1 and mTOR (Fig. 1A). In addition, the anti-mTOR antibody also pulled down NBS1 (Fig. 1B), demonstrating their interaction when both proteins were overexpressed in 293T cells. Similar assays were performed to test the interaction between NBS1 and Rictor and the results showed that NBS1 interacted with Rictor in 293T cells overexpressing both proteins (Fig. 1C–D). Finally, similar assays were performed to test the interaction between NBS1 and SIN1β, which also showed the interaction between these two proteins (Fig. 1E–F). All the results demonstrated that NBS1 interacted with the mTOR/Rictor/SIN1 complex.

### Mapping of Domain in mTOR or Rictor Interacting with NBS1

**Figure 2:**
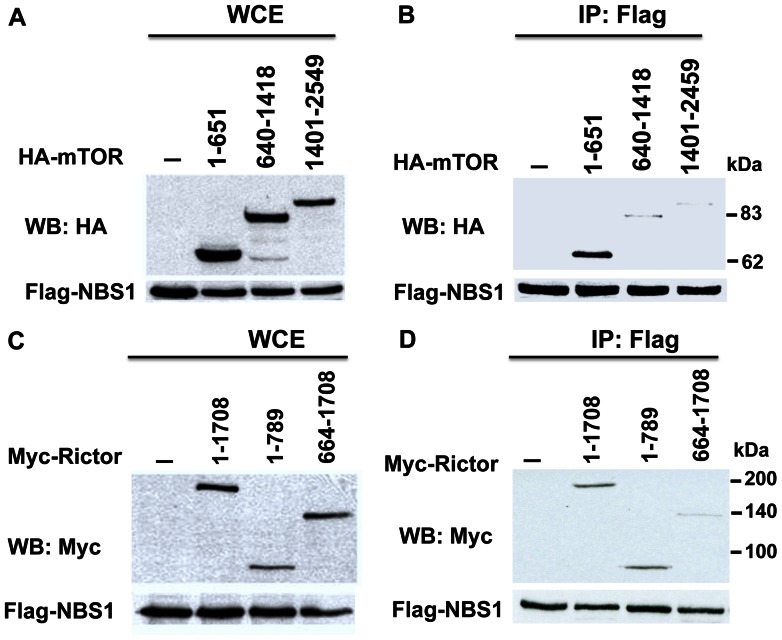
Mapping of the domain in mTOR or Rictor interacting with NBS1. **A & B.** Co-immunoprecipitation assays showed the interaction between the N-terminal domain of mTOR and NBS1. Whole cell extracts were shown in A. **C & D.** Co-immunoprecipitation assays showed the interaction between the N-terminal domain of Rictor and NBS1. Whole cell extracts were shown in C.

In order to map the domain in mTOR or Rictor that interacts with NBS1, different truncation mutants of mTOR or Rictor were generated. Different truncation mutants of mTOR (mTOR1-651, mTOR640-1418, and mTOR1401-2549) were co-expressed with NBS1 in 293T cells followed by co-immunoprecipitation. The results showed that only mTOR1-651 interacted with NBS1 (Fig. 2A–B). The full length and different truncation mutants of Rictor (Rictor1-1708, Rictor1-789, and Rictor664-1708) were co-expressed with NBS1 in 293T cells followed by co-immunoprecipitation. Co-immunoprecipitation experiments showed that the domain 1-789 a.a. of Rictor interacted with NBS1 (Fig. 2C–D). These results indicated that the N-terminal domains (1-651 a.a. domain of mTOR and 1-789 a.a. domain of Rictor) of both proteins interacted with NBS1.

### Mapping of the NBS1 Domain Interacting with mTOR or Rictor

**Figure 3:**
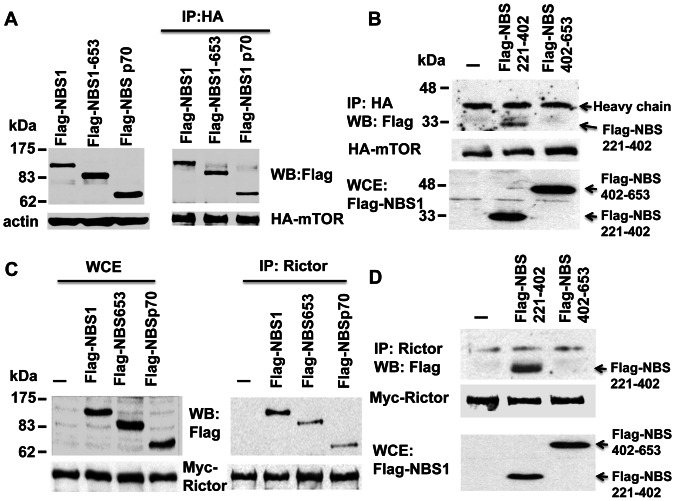
Mapping of the domain in NBS1 interacting with mTOR or Rictor. **A & B.** Co-immunoprecipitation assays showed the interaction of the internal domain (a.a. 221-402) of NBS1 with mTOR. **C & D.** Co-immunoprecipitation assays showed the interaction of the internal domain of NBS1 with Rictor.

In order to map the domain in NBS1 that interacts with mTOR or Rictor, different truncation mutants of NBS1 (C-terminal truncation mutant NBS1-653 and N-terminal truncation mutant NBSp70) were co-expressed with mTOR or Rictor followed by co-immunoprecipitation [12]. The result showed that both the mutants interacted with mTOR and Rictor (Fig. 3A, C). Further fine mapping of the domain using two different NBS1 truncation mutants (NBS221-402 and NBS402-653) together with mTOR or Rictor showed that only NBS221-402 interacted with mTOR and Rictor (Fig. 3B, D). All the results indicated that the internal domain (a.a. 221-402) of NBS1 interacted with both mTOR and Rictor.

### Mapping of the Interacting Domain between NBS1 and SIN1β

**Figure 4:**
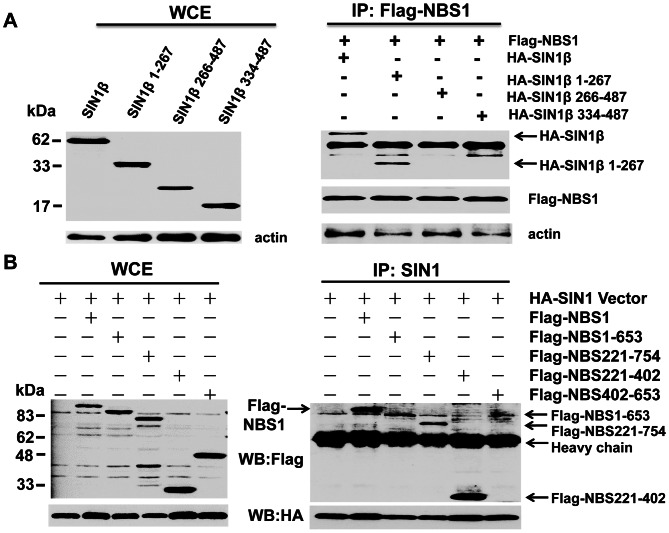
Mapping of the domain of SIN1β or NBS1 interacting with each other. **A.** Co-immunoprecipitation assays showed the interaction of the N-terminal domain (a.a. 1-267) of SIN1β with NBS1. **B.** Co-immunoprecipitation assays showed the interaction of the internal domain (a.a. 221-402) of NBS1 with SIN1β.

To map the domain in SIN1β that interacts with NBS1, different SIN1β truncation mutants (SIN1β1-267, SIN1β266-487, SIN1β336-487) were co-expressed with NBS1 in 293T cells followed by co-immunoprecipitation. The result showed that only SIN1β1-267 interacted with NBS1 (Fig. 4A). To map the domain in NBS1 interacting with SIN1β, different NBS1 truncation mutants as mentioned above were co-expressed with SIN1β in 293T cells followed by co-immunoprecipitation. The result showed that the domain in NBS1 interacting with SIN1β was also mapped to the a.a. 221-402 domain of NBS1 (Fig. 4B).

### *In vivo* Interaction between NBS1 and the mTOR/Rictor/SIN1 Complex

**Figure 5:**
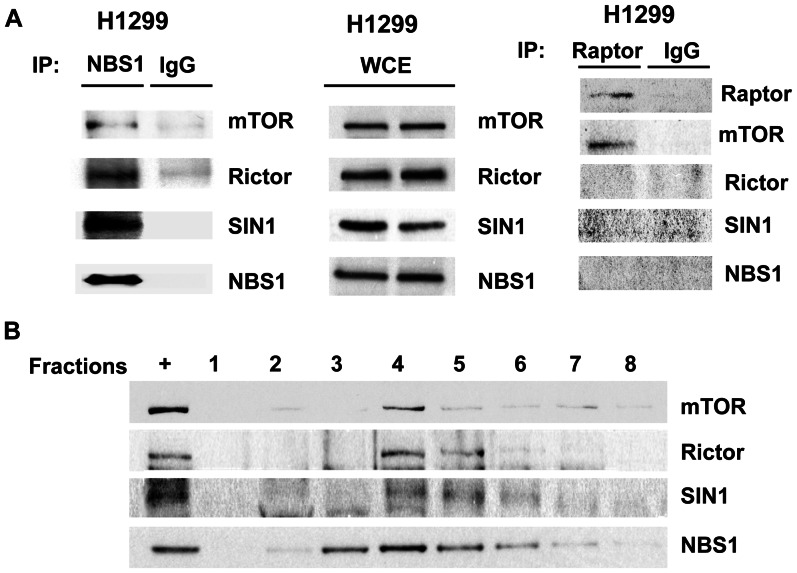
Co-immunoprecipitation assays and sucrose density gradient analysis showed the interaction between NBS1 and the mTOR/Rictor/SIN1 complex *in vivo*. **A.** Co-immunoprecipitation assays showed the *in* *vivo* interaction of NBS1 with the mTOR/Rictor/SIN1 complex using extracts from H1299 cell line. **B.** Sucrose density gradient analysis showed the co-localization in the same fractions between NBS1 and the mTOR/Rictor/SIN1 complex.

In order to test whether NBS1 indeed interacts with the mTOR/Rictor/SIN1 complex, co-immunoprecipitation assays using extracts from a lung cancer cell line H1299 were used. The result showed that the anti-NBS1 antibody pulled down mTOR, Rictor, and SIN1β (Fig. 5A, left panel), supporting their interaction *in vivo*. Co-immunoprecipitation experiment using the anti-Raptor antibody did not pull down the whole mTOR/Rictor/SIN1 complex or NBS1 (Fig. 5A, right panel), indicating that NBS1 did not interact with the mTOR/Raptor complex. Finally, a sucrose density gradient experiment using extracts from H1299 cells was performed to test whether these proteins were localized in the same fraction. The result showed that indeed NBS1, mTOR, Rictor, and SIN1β were localized in the same fraction (fraction 4 is the major fraction) (Fig. 5B). All the results indicated that NBS1 interacted with the mTOR/Rictor/SIN1 complex *in* *vivo*.

### Knockdown of NBS1 Decreased the Levels of Phosphorylated Akt and its Downstream Targets

**Figure 6:**
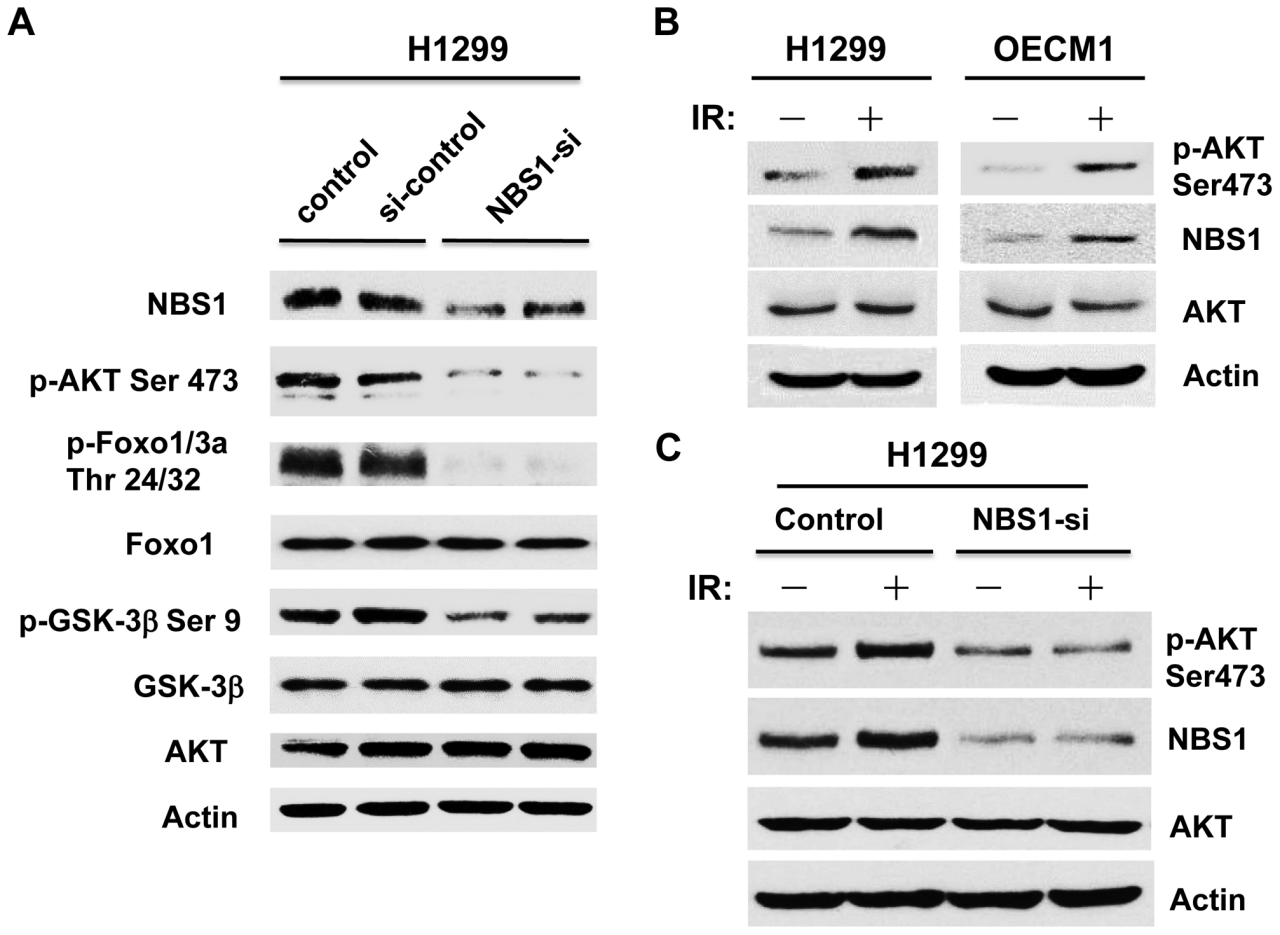
siRNA mediated knockdown of NBS1 decreased the levels of phosphorylated Akt (pAkt Ser-473) and its downstream targets and IR increased the levels of NBS1 and phosphorylated Akt. **A.** Western blot analysis showed that knockdown of NBS1 decreased the phosphorylated levels of Akt, GSK-3β, and Foxo1/3a). **B.** Ionizing radiation of two different cell lines (H1299, OCEM-1) increased the levels of NBS1 and phosphorylated Akt. **C.** Knockdown of NBS1 in H1299 cells abolished the increase in NBS1 and phosphorylated Akt levels under IR.

In order to test the correlation between NBS1 levels and its effect on the Akt activity, siRNA experiment to knockdown NBS1 was performed. The result showed that knockdown of NBS1 decreased the phosphorylated Akt levels (pAkt Ser-473) (Fig. 6), indicating that NBS1 contributed to the Akt activity. In addition, the phosphorylation levels of certain Akt downstream targets such as GSK-3β and Foxo1/3a were also decreased following NBS1 knockdown (Fig. 6A). These results supported the role of NBS1 in the activation of Akt activity.

### Ionizing Radiation (IR) Increased the NBS1 Levels and Activated Akt

Since IR was shown to activate Akt signaling [23], [24], we tested the physiological relevance of NBS1 to activate Akt activity through IR. IR increased the levels of NBS1 and phosphorylated Akt (pAkt Ser-473) in two different cell lines (Fig. 6B). Knockdown of NBS1 in H1299 cells abolished the increase in the levels of NBS1 and phosphorylated Akt under IR treatment (Fig. 6C). These results supported the physiological role of NBS1 to activate Akt activity.

## Discussion

Overexpression of NBS1 was previously shown to induce transformation through the PI 3-kinase/Akt pathway [11]–[13]. However, in this report, we demonstrated that NBS1 overexpression could also interact with the mTOR/Rictor/SIN1 complex to activate Akt activity. This result is the further extension of the ability of NBS1 to interact with both the upstream PI3K and downstream Akt to regulate the activity of PI3K/Akt pathway. It is also supported by recent literatures demonstrating the dual function of NBS1 in the repair of DNA breaks and proliferation and the requirement of NBS1 in IGF-1 induced cellular proliferation [25], [26]. In addition, the ability of RNA regulon to promote Akt activity also goes through the upregulation of NBS1 levels [27]. Both results support the role of NBS1 in the activation of Akt activity.

SIN1 was the critical molecule recently demonstrated to be important for the activation of Akt activity through interacting with the mTOR/Rictor complex [19]–[21]. Other molecules that could interact with the mTOR/Rictor complex were also recently identified including PRR5 and the TSC1/TSC2 complex [22], [28]. The ability of NBS1 to interact with each component of the mTOR/Rictor/SIN1 complex showed that NBS1 may be another critical component of the mTOR/Rictor/SIN1 complex, which was supported by the result that knockdown of NBS1 decreased the phosphorylation levels of Akt and its downstream targets (Fig. 6A). In addition, knockdown of NBS1 abolished the increase in NBS1 and phosphorylated Akt levels under IR, supporting the physiological relevance of NBS1 in the activation of Akt activity (Fig. 6B–C). This result also points to the role of a DNA double strand break repair protein to activate Akt activity. It is interesting that NBS1 uses the same internal domain (a.a. 221-402) to interact with each component of the mTOR/Rictor/SIN1 complex. This domain contains the ATM-dependent phosphorylation sites (Ser278, Ser343, Ser397). In contrast, NBS1 uses the conserved C-terminal domain (a.a. 653-669) to interact with the subunits of PI3K to achieve its activation [12]. Further structural analysis of co-crystalized NBS1 and the mTOR/Rictor/SIN1 complex will delineate the molecular interactions between these molecules. Our previous results showed that increased NBS1 expression was constantly observed in different tumor samples [11], [13], [29], suggesting that increased NBS1 levels in tumor tissues may contribute significantly to the increased Akt activity observed in different tumor samples. Our discovery points to the unique role of NBS1 in the activation of the PI3K/Akt pathway using a newly identified internal domain (a.a 221-402) to interact with the mTOR/Rictor/SIN1 complex to achieve activation.

## Materials and Methods

### Cell Lines, Plasmids, Transfections, and Stable Clones

The human embryonic kidney 293T and H1299 (a non-small cell lung cancer cell line) were previously described [12], [30]. The OCEM-1 cell line is a head and neck cancer line obtained from ATCC. The Flag-NBS1 full length and its truncation mutants were described [12]. The pSUPER-NBS1 and pSUPER-scrambled-si constructs were described [12]. The full length HA-mTOR expression vector was constructed by assembling fragments containing the nucleotides 1-4254 and 4201-7650 of the *mTOR* gene that were amplified from the human Marathon-Ready brain cDNA (Clontech) by PCR and subcloned into the yTA (Yeastern) vector. The sequences of primers used were 5′-CAATTGGTCGACATGCTTGGAACCGGACCTGC-3′ and 5′-GCTAGCGCGCTAGCTGATGAGAGATTCTAGAATGG-3′ for *mTOR* (1-4254), and 5′-GAACTGGAGTTCCAGTTCCAGAAAGGC-3′ and 5′-CAATTGGCGCGCTTACCAGAAAGGGCACCAGC-3′ for *mTOR* (4201-7650).

The yTA-mTOR(1-4254) and yTA-mTOR(4201-7650) plasmids were digested by *Sal*I and *BssH*II and by *Xba*I and *BssH*II, respectively. Each mTOR fragment was ligated into the pDNR-CMV vector (Clontech). The SalI-XbaI mTOR fragment from the pDNR-CMV-mTOR(1-4254) plasmid was ligated into the pDNR-CMV-mTOR(4201-7650) plasmid to generate the full length pDNR-CMV-mTOR(1-7650) expression vector. The HA epitope-tagged full-length mTOR was generated following the BD Creator cloning protocol (Clontech) using the pLP-CMV-HA vector as the acceptor plasmid. The Myc-Rictor expression vector was generated by assembling PCR fragments amplified from human Marathon-Ready brain cDNA (Clontech) to make the full-length *Rictor* expression vector. The ATG-containing 5′ primer and the stop codon-containing 3′ primer (described in Table 1) introduced *Sal*I and *Kpn*I restriction sites, respectively. The final assembled fragment of *Rictor* (nucleotides 1-5191) was digested by the two endonucleases and ligated into the pCMV-Myc (Clontech) plasmid to generate the full length pCMV-Myc-Rictor (1-5191) expression vector. The HA-mTOR and Myc-Rictor truncation mutants were constructed by using the appropriate primers and PCR to generate different truncation mutants [31], [32] (Table 1). Since there are different SIN1 splice variants [33], only the SIN1β full length expression vector was generated by using RT-PCR of mRNA from 293T cells followed by inserting into the CMV-HA vector. The oligonucleotides used to generate the full length SIN1β and its truncation mutants were also described in Table 1.

**Table 1:** The oligonucleotide sequences used to generate various full length and truncation constructs of mTOR, Rictor, and SIN1β.

DNA constructs	Sequence (5′→3′)	Restriction Enzyme site	Ampli-con (bp)
**mTOR constructs**			
pCMV-HA-mTOR_1-2549_ (full length)	F:CAATTGGTCGACATGCTTGGAACCGGACCTGC	*Mfe*I, *Sal*I	7674
	R:CAATTGGCGCGCTTACCAGAAAGGGCACCAGC	*Mfe*I, *BssH*II	
pCMV-HA-mTOR_1-651_	F: CAATTGGTCGACATGCTTGGAACCGGACCTGC	*Mfe*I, *Sal*I	1977
	R:GCTAGCGCGCTACACTTGCACTGCGGTCTGGC	*Nhe*I, *BssH*II	
pCMV-HA-mTOR_640-1418_	F: CAATTGGTCGACCATGCTCATGTGGTTAGCC	*Mfe*I, *Sal*I	2361
	R:GCTAGCGCGCTAGCTGATGAGAGATTCTAGAATGG	*Nhe*I, *BssH*II	
pCMV-HA-mTOR_1401-2549_	F: GAACTGGAGTTCCAGAAAGGC	*Xba*I from vector	3466
	R: CAATTGGCGCGCTTACCAGAAAGGGCACCAGC	*Mfe*I, *BssH*II	
**Rictor constructs**			
pCMV-Myc-Rictor_1-1708_ (full length)	F:GATTGACGTCGACCGTCAATATGGCGGCGATCG	*Sal*I	5170
	R:CTGGTACCATCATAAATATGAGGTCAGGATTC	*Kpn*I	
pCMV-Myc-Rictor_1-789_	F:GATTGACGTCGACCGTCAATATGGCGGCGATCG	*Sal*I	2407
	R:CTGGTACCTACTGAATGAGAGCATGAAGATTGGC	*Kpn*I	
pCMV-Myc-Rictor_664-1708_	F: TGCGTCGACTGGAACACTTTCTTGCCACCCTC	*Sal*I	3171
	R: CTGGTACCATCATAAATATGAGGTCAGGATTC	*Kpn*I	
**SIN1β constructs**			
pCMV-HA-SIN1β	F: CGGAATTCCGGCCTTCTTGGACA	*EcoR*I	1461
(full length)	R: GAAGATCTTCACTGCTGCCCGG	*Bgl*II	
pCMV-HA-SIN1β_1-267_	F: CCGGAATTCGGATGGCCTTCTTGGAC	*EcoR*I	801
	R: CGGGGTACCTCACTTTTCAACCAGGGC	*Kpn*I	
pCMV-HA-SIN1β_266-486_	F: CCGGAATTCGGGAAAAGTACTCATCT	*EcoR*I	666
	R: CGGGGTACCTCACTGCTGCCCGGA	*Kpn*I	
pCMV-HA-SIN1β_334-486_	F: CCGGAATTCGGGACATAGCCACAGTA	*EcoR*I	462
	R: CGGGGTACCTCACTGCTGCCCGGA	*Kpn*I	

### Western Blot Analysis and Co-immunoprecipitation Assays

Western blot analysis was performed as described [12]. For Western blot analysis, 50 µg protein extracts from each clone were loaded to 10% SDS-PAGE gels and transferred to nitrocellulose filters. The filters were probed with various antibodies (described in Table 2) and an anti-ß-actin antibody was selected as a loading control. Signals were developed using an ECL chemiluminescence kit (Amersham Biosciences, U.K.).

**Table 2:** List of proteins and characteristics of the corresponding antibodies used to detect the proteins.

Protein	Assay	Antibody	Origin	Dilution	Incubation period
NBS1	WB	rpab	#3002, Cell Signaling Technology	1∶1000	Overnight
NBS1	WB	rpab	NB100-143, Novus Biologicals	1∶10000	Overnight
NBS1	IP	rpab	NB100-143, Novus Biologicals	1.0 µl	Overnight
mTOR	WB	rpab	#2972, Cell Signaling Technology	1∶1000	Overnight
mTOR	IP	rpab	#2972, Cell Signaling Technology	5.0 µl	Overnight
Rictor	WB	mmab	H00253260-M01, Abnova	1∶1000	Overnight
Rictor	IP	mmab	H00253260-M01, Abnova	5.0 µl	Overnight
SIN1	WB	rpab	A300-910A, Bethyl Laboratories	1∶2000	Overnight
Flag	WB	mmab	F3165, Sigma-Aldrich	1∶1000	1 hour
Flag	IP	mmab	F3165, Sigma-Aldrich	5.0 µl	Overnight
His-tag	WB	mmab	H1029, Sigma-Aldrich	1∶10000	Overnight
Myc-tag	WB	mmab	LTK Biolaboratories	1∶500	Overnight
Akt	WB	rpab	#9272, Cell Signaling Technology	1∶1000	Overnight
p-Akt	WB	rpab	#9271, Cell Signaling Technology	1∶1000	Overnight
p-GSK-3β	WB	rpab	#9336, Cell Signaling Technology	1∶1000	Overnight
p-Foxo1/3a	WB	rpab	#9464, Cell Signaling Technology	1∶1000	Overnight
GSK-3β	WB	rmab	#9315, Cell Signaling Technology	1∶1000	Overnight
Foxo1	WB	rpab	#9454, Cell Signaling Technology	1∶1000	Overnight
Actin	WB	mmab	A5441, Sigma-Aldrich	1∶20000	1 hour

Abbreviations: WB, Western blot; IP, Immunoprecipitation; mmab, mouse monoclonal antibody; rpab, rabbit polyclonal antibody.

Co-immunoprecipitation assays were performed as described [12], [34]. Briefly, the anti-NBS1, anti-mTOR, anti-Rictor, anti-SIN1, anti-HA, or anti-Myc antibody was incubated for 5 h with 500 µl of whole cell extracts prepared by lysis in 150 mM NaCl, 1% Nonidet P-40, 1% deoxycholate, 0.1% SDS, 50 mM Tris.HCl-pH 7.5, and protease inhibitors, from 293T cells overexpressing proteins of interest or from H1299 cells to detect *in* *vivo* interaction. The immune complexes were incubated overnight with protein-A beads, preblocked with 10% BSA. The immunoprecipitates were washed 3 times with TNTG buffer (20 mM Tris.HCl-pH 7.5, 150 mM NaCl, 0.1% Triton-X 100, 10% glycerol), mixed with 1x Laemmli dye, boiled for 10 min and loaded on SDS- polyacrylamide (SDS-PAGE) gels [12]. After transfer, the filters were blocked with blocking buffer [12], probed with primary and secondary antibody sequentially and developed. The antibodies used are described in Table 2. Data shown are representative of at least 2 experiments from independent cell cultures or different transfections.

### Sucrose Density Gradient Analysis

A sucrose density gradient was generated as described [35] and the extracts of H1299 cells were loaded to the continuous gradient. After centrifugation, the proteins were collected from different fractions, run on SDS-PAGE, and transferred to membranes. The blot was probed with various antibodies to detect the presence of different components of mTOR/Rictor/SIN1 and NBS1.

### Ionizing Radiation (IR)

Exponentially growing cells were temporarily incubated with 2 ml of PBS in 10 cm dishes and were irradiated at room temperature using ^60^Co γ-ray source with a dose of 5.0 Gy (dose rate 3 Gy/min). After irradiation, cells were washed twice with PBS and cultured in fresh DMEM medium containing 10% FBS for another 12 hours followed by harvesting for Western blot analysis.
